# Photoelectrocatalytic hydrogen generation coupled with reforming of glucose into valuable chemicals using a nanostructured WO_3_ photoanode

**DOI:** 10.1038/s42004-022-00745-w

**Published:** 2022-10-13

**Authors:** Katarzyna Jakubow-Piotrowska, Bartłomiej Witkowski, Jan Augustynski

**Affiliations:** 1 Centre of New Technologies, University of Warsaw, S. Banacha 2c, 02-097 Warsaw, Poland; 2 Faculty of Chemistry, University of Warsaw, Pasteura 1, 02-093 Warsaw, Poland

**Keywords:** Photochemistry, Electrochemistry

## Abstract

Coupling the photo-oxidation of biomass derived substrates with water splitting in a photoelectrochemical (PEC) cell is a broadly discussed approach intended to enhance efficiency of hydrogen generation at the cathode. Here, we report a PEC device employing a nanostructured semitransparent WO_3_ photoanode that, irradiated with simulated solar light achieves large photocurrents of 6.5 mA cm^−2^ through oxidation of glucose, a common carbohydrate available in nature that can be obtained by processing waste biomass. The attained photocurrents are in a large part due to the occurrence of the photocurrent doubling, where oxidation of glucose by the photogenerated positive hole is followed by injection by the formed intermediate of an electron into the conduction band of WO_3._ Selection of an appropriate supporting electrolyte enabled effective reforming of glucose into valuable products: gluconic and glucaric acids, erythrose and arabinose with up to 64% total Faradaic yield attained at ca 15% glucose conversion.

## Abstract (web summary)

Coupling the photo-oxidation of biomass-derived substrates with water splitting in a photoelectrochemical cell enables efficient hydrogen generation at the cathode. Here, a photoelectrochemical device employing a nanostructured WO_3_ photoanode displays photocurrents of 6.5 mA cm^−2^ through oxidation of glucose, in turn producing valuable products in the form of gluconic and glucaric acids, erythrose and arabinose.

## Introduction

The continuing research on photoelectrochemical (PEC) water splitting to allow generation of sustainable hydrogen fuel from water and sunlight is now principally oriented towards improving the stability and performance of the employed photoelectrodes^1–3^. In the most frequently employed PEC cell configuration, an n-type semiconductor (SC) photoanode (where photogenerated positive holes, h^+^, oxidize water to form O_2_) is combined with a metallic cathode (where electrons, e^-^, transferred from the photoanode, reduce protons to form H_2_). It is to be noted, in this connection that, considering high overpotentials accompanying the oxygen evolution reaction (OER) on conventional (unilluminated) anodes, the SC photoanodes may arguably offer larger advantage than the photocathodes for the performance of a PEC water splitting cell. As regards the photoanodes, the only materials able to photo-oxidize water whilst avoiding photocorrosion (essentially for kinetic reasons) are metallic oxides^4,5^, and only few of them (Fe_2_O_3_, BiVO_4_, WO_3_) feature optical absorption spectra that match terrestrial solar spectrum. Since none among those photostable - metallic oxide - semiconductors exhibits band-edge energy levels that match those of hydrogen and oxygen evolution reactions to allow unassisted water splitting, the present efforts focus on minimizing the bias voltage allowing the photoanode to reach plateau (saturation) photocurrent when performing visible light-driven photo-oxidation of water^6–11^. In fact, the moderate operating potential point at which the photocurrent already reaches saturation is a precondition for combining the photoelectrolyzer with a photovoltaic (PV) cell to form a tandem device^12–14^. Actually, a bias voltage of the order of 1 V (at which the semitransparent WO_3_ photoanode approaches saturation photocurrent) may be provided by several single-junction photovoltaic (PV) cells likely to operate in a tandem device with the PEC cell.

The principal strategy developed for improving the photocurrent-voltage (j-E) behavior of the SC photoanodes, by reducing e^-^-h^+^ recombination losses, involved deposition on the photoanodes of OER electrocatalysts such as mixed cobalt phosphate-oxide (Co–Pi)^15,16^ or oxyhydroxides of nickel and iron^17,18^.

To address this issue, several authors also attempted to combine water splitting with the kinetically easier photo-oxidation of biomass derivatives to replace or complement oxygen evolution on the photoanode. However, an optimal implementation of such concept would require, rather than total mineralization at the photoanode of the used biomass-derived reagent (to form carbon dioxide), its efficient conversion into value-added products. Early studies aimed to enhance hydrogen production at the cathode via photo-reforming at the anode of biomass-derived products used principally nano-crystalline (NC) TiO_2_ photoanodes^19–22^ and ethanol as model compound acting as a hole scavenger. When an alkaline electrolyte (1 M NaOH) was employed by the authors^19,20,22^ in the anode compartment, the ethanol solutions did not reach total mineralization due, in particular, to the formation of insoluble aldol condensation products - high molecular weight aldehydes^22^. The latter investigations followed parallel studies in this area that used relatively low concentrations of oxygenate biomass derivatives with suspended in the solutions TiO_2_ nanoparticles (NPs) acting as photocatalysts^23,24^.

However, an important advantage of the PEC cell configuration is in the facility of produced gas separation and the possibility of controlling the atmosphere in the anode compartment. Although the photo-oxidation of biomass-derived oxygenates at NC TiO_2_ photoanodes has been shown to be highly efficient^25^ and to benefit, in addition, from the photocurrent doubling effect^26^, the large band gap of the semiconductor (E_g_ = 3.2 eV), for the anatase form (that is the major component of the widely employed commercial P25 particles) is an evident limitation for the effective use of sunlight as the irradiation source.

The photocurrent doubling effect has also been demonstrated for the photo-oxidation of organic C1^27^ and larger^28^ molecules at nanostructured (NS) tungsten trioxide photoelectrodes that, due to their lower bandgap energy (E_g_ = 2.5–2.6 eV), are capable to also absorb light over the visible blue portion of the solar spectrum. Despite the fact that E_g_ of WO_3_ is larger than the optimum value of 2-2.2 eV, the NS WO_3_ photoanodes attained considerable photocurrent efficiencies^29,30^ due to relatively long hole diffusion length of ca 150 nm^31,32^ that reduces bulk electron-hole recombination within the NPs that form the film. On the other hand, the efficient photogenerated current collection at the electrode substrate is favored by large (in the range of 6.5-12 cm^2^ V^−1^ s^−1^) electron mobility in the crystalline WO_3_^33,34^.

A more recent work^35^, employing thin film WO_3_ photoanodes formed by sputtering confirmed the occurrence of the photocurrent doubling also in the case of photo-oxidation of a biomass-derived monosaccharide – glucose. Significant information that stemmed from that study was a relatively low extent of glucose conversion into CO_2_, in particular in more concentrated (0.1 M) solutions, that indicated the dominant formation of intermediate oxidation products that could not been identified. We note in this connection that numerous experiments on photo-reforming glucose and/or lignocellulose using suspensions of TiO_2_ photocatalysts loaded with noble metal’s (most frequently Pt) NPs^36–39^, in which changes in the solution composition were monitored using high-performance liquid chromatographic (HPLC) analyses, showed the formation of a wide variety, some of them high-value, oxidation products. In one of such studies, the TiO_2_ doping with tungsten allowed the operation of such Pt-TiO_2_(W) photocatalyst under sunlight irradiation while TiO_2_ doping with nitrogen favored formation, besides H_2_ and CO_2_, of arabinose, erythrose and formic acid as partial glucose oxidation products^37^.

An interesting approach, intended to avoid the use of noble metal catalysts required to reduce protons into H_2_ in the case of photo-reforming of lignocellulose at TiO_2_ photocatalysts^39^, resulted in the development of a photocatalytic system based on n-type SC cadmium sulfide quantum dots^40^. In fact, CdS (E_g_ = 2.4 eV) that absorbs the blue portion of the visible spectrum features the conduction band (CB) level at a potential sufficiently negative (ca −0.5 V vs SHE) to favor H_2_ evolution in the absence of co-catalysts. To conduct photo-reforming of α-cellulose, the authors used a strongly alkaline (10 M KOH) solution, the conditions under which the surface of suspended CdS quantum dots became passivated by a CdO_x_ overlayer^40^. However, despite efficient sunlight-driven H_2_ evolution, due to the complex chemistry involved in the synthesis of CdS quantum dots and the well-known toxicity of cadmium compounds, the latter work may be rather considered as an interesting proof of concept.

Among few examples in which upgrading of biomass-derived compounds was performed in a PEC cell, and not in a photocatalytic system featuring suspension of SC particles^41^, is the oxidation on a BiVO_4_ - based photoanode of an important intermediate in biomass conversion 5-hydroxymethylfurfural (HMF) into 2,5-difurancarboxylic acid (FDCA) - a monomer used for the synthesis of several polymers^42^. The advantage of the employed in that case PEC configuration is that hydrogen was co-generated in a separate cathode cell compartment.

Herein we describe a series of experiments performed in a PEC cell featuring NS WO_3_ photoanodes, in which we aimed to optimize the photocurrents generated through photo-oxidation of glucose, selected as the target compound, in order to enhance the efficiency of hydrogen generation at the cathode. Let us to recall that glucose is a common carbohydrate available in nature that can be obtained through the hydrolysis of cellulose and hemicellulose present in waste biomass. We demonstrate that a significant replacement of OER with the photo-oxidation of glucose results in a large increase of the photocurrents attained at the NS WO_3_ photoanode due to the more favorable reaction kinetics and, especially, due to the occurrence of the photocurrent doubling. We probed the photocurrent efficiency as well as stability of the photoanode to also find out the most appropriate electrolyte composition, which allows the formation of the largest amount of valuable organic products – gluconic and glucuronic acids, arabinose and erythrose.

## Results

### Preparation and structural characterization of WO_3_ film electrodes

We synthesized the NS WO_3_ films by a sol-gel method involving layer-by-layer deposition on the substrate of a colloidal solution of freshly prepared tungstic acid, complemented by the addition of polyethylene glycol (PEG 300), followed by high temperature (550-600 °C) annealing in oxygen. Conductive fluorine-doped tin oxide (FTO)-coated glass sheets served as electrode substrates. As confirmed by repeated PEC experiments performed with the same electrodes, the use of the sequential layer-by-layer precursor deposition/annealing procedure is essential for building structurally stable, relatively thick (up to 3 μm thick) WO_3_ films with good adherence to the substrate. A single standard application of the precursor produced typically a ~0.4 μm thick WO_3_ film. The films used in this work were formed by three-to-eight consecutive applications of the precursor solution, each followed by the high-temperature annealing in O_2_ for 30 min. See Methods for further details.

**Fig. 1:**
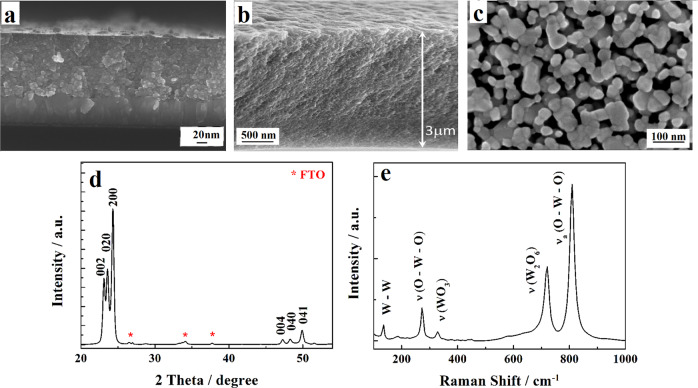
Morphological and structural characterization of NS tungsten trioxide electrodes. **a** and **b**, Cross-sectional SEM images of ~ 1.2 μm (**a**) and ~ 3 μm (**b**) thick WO_3_ films. **c**, top-down SEM image of the film in (**a**). **d**, X-ray diffraction pattern of WO_3_ film in (**a**). **e**, Raman spectrum of WO_3_ film in (**a**).

As shown by the cross-sectional scanning electron microscopic (SEM) image of a ~1.2 μm thick WO_3_ film in Fig. 1a, the employed sol-gel method produces a mesoporous film with some pores extending apparently down to the FTO substrate. The significant porosity is even better perceived on the SEM image of a thinner 0.4 μm thick WO_3_ film (Supplementary Fig. S1). We consider the formation of such a porous film being the result of burning the PEG, present in the precursor, occurring during the high-temperature annealing in oxygen. We also presume that the local heating involved in the latter process allows to establish crystalline interparticle contacts^43^ and contributes to the stability of the formed mesoporous film. The top-down surface SEM image in Fig. 1c shows that the film consists of a network of small individual, ~20–40 nm in size, NPs as well as of multiparticle agglomerates.

The annealing at a temperature of 550 °C or above it results in the formation of WO_3_ films with the well-defined monoclinic crystal structure. X-ray diffraction (XRD) pattern (Fig. 1d) exhibits three main sharp peaks (002), (020), (200) with three other less intense (004), (040) and (041) peaks - consistent with the preferential orientation of the WO_3_ crystallites parallel to the substrate plane (COD - Crystallography Open Database # 96-210-6383). Also the Raman spectrum of the formed film (Fig. 1e) shows the principal features characteristic of the monoclinic structure of WO_3_ with prominent bands at around 715 and 805 cm^−1^.

### Photoelectrochemical characterization

Concomitant with the use of WO_3_ film as a photoanode is the choice of supporting electrolyte - the most appropriate to perform the PEC oxidation of the chosen organic compound. As initially reported^44^ and subsequently confirmed in a series of works^45–47^, the WO_3_ photoanodes have a pronounced tendency to favor oxidation of anions of the acidic electrolytes, the reactions that in some cases (H_2_SO_4_, HClO_4_) are accompanied by the formation of the peroxide species that, over prolonged water splitting experiments, causes progressive electrode deactivation. As demonstrated by Raman spectroscopic analyses of the H_2_SO_4_ electrolytes, submitted to several hours long photoelectrolysis, the peroxodisulfates (S_2_O_8_^2-^) become in that case the main product formed at the WO_3_ photoanode^44^.

However, an important exception is the excellent photostability of the WO_3_ electrodes in electrolytes, including chlorides with the dominant chlorine evolution occurring at the photoanode^44,48^. Another exception is the behavior of WO_3_ photoanodes in methanesulfonic acid (CH_3_SO_3_H) electrolyte in which stable oxygen evolution photocurrents are observed^48,49^ that we assign to high reactivity with water of the initially formed, CH_3_SO_3_^·^ radical species^50^. The photo-oxidation products of the anions of acidic supporting electrolytes, including the corresponding radical species, are expected to play a role of intermediates in the PEC reactions involving organic substrates especially when they are present at low concentrations^28^.

**Fig. 2:**
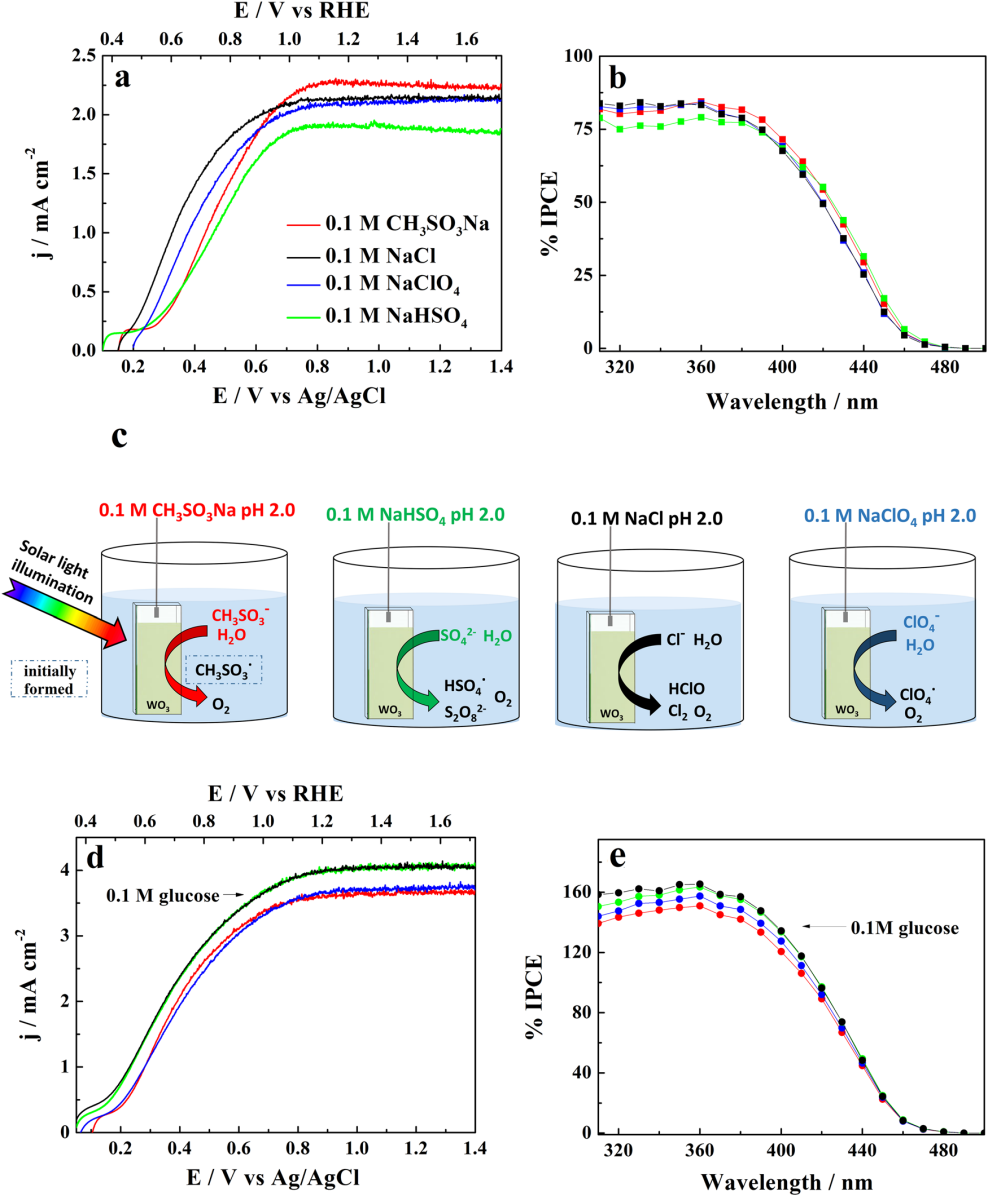
Photoelectrochemical glucose oxidation performance. **a** Photocurrent densities vs imposed potential (j-E) plots recorded under simulated AM 1.5 G irradiation and **b**, The corresponding incident photon-to-current efficiency (IPCE) spectra measured at 1.23 V vs RHE for a ~1.2 μm thick film WO_3_ electrode in a series of 0.1 M supporting electrolytes acidified to pH 2. **c** The photo-oxidized species formed on the WO_3_ photoanode in the employed electrolytes. **d** Effect of the addition of 0.1 mol L^−1^ of glucose upon j-E plots recorded for the electrolytes listed in (**a**). **e**, The corresponding IPCE spectra.

In order to compare the effect of different supporting electrolytes upon glucose photo-oxidation efficiency, we used a three-electrode cell configuration with a Pt cathode separated from the WO_3_ photoanode by a Nafion membrane and an Ag/AgCl reference electrode (see Methods). For those preliminary screening tests we employed relatively thin (~1.2 μm thick) WO_3_ film photoanodes. The initially used electrolytes consisted of moderately concentrated 0.1 M solutions of sodium chloride, perchlorate, sulfate and methanesulfonate acidified to pH 2 that remains in the range of thermodynamic WO_3_ stability extending up to pH 4–5. In Fig. 2 are represented linear sweep voltammograms (j-E) recorded for WO_3_ photoelectrodes in contact with supporting electrolytes alone (Fig. 2a) and, subsequently, after addition to the anodic compartment of 0.1 mol L^−1^ of glucose (Fig. 2d). In both cases the photoelectrodes were irradiated with simulated solar light (AM 1.5 G of 100 mW cm^−2^ intensity). The voltametric scans recorded in supporting electrolytes showed photocurrent onset potentials at ~0.15 V versus Ag/AgCl (corresponding to 0.47 V vs reversible hydrogen electrode (RHE)) and reached the plateaus of around 2.2 mA cm^−2^, at ~0.8 V vs Ag/AgCl. The largest plateau photocurrent was observed in a CH_3_SO_3_Na electrolyte and a slightly lower in NaHSO_4_, apparently indicative of the formation of peroxodisulfate species on the WO_3_ surface. It is to be noted that these photocurrents are limited by the restrained optical thickness of the WO_3_ photoanodes that we used in those screening test.

Addition to the supporting electrolytes of 0.1 mol L^−1^ of glucose led to a strong increase of the recorded photocurrents (Fig. 2d) consistent with the occurrence at the WO_3_ electrode of the photocurrent doubling^26,27^. We wish to mention that the use, in this context, of the term photocurrent doubling implies, in fact, an important contribution of the mechanism where oxidation of glucose by the photogenerated positive hole, h^+^, is followed by the injection, by the formed reaction intermediate, of an electron into the CB of WO_3._ The largest increase of the photocurrents was observed in the solutions containing acidic sulfate and chloride electrolytes to achieve plateaus of ~4.2 mA cm^−2^. As shown in Fig. 2d, the photocurrents set in at about 0.1–0.15 V more negative potentials suggesting that the presence of glucose also significantly diminished the extent of charge carrier e^-^ - h^+^ recombination. The corresponding dark currents measured in the supporting electrolytes and with added 0.1 mol L^−1^ of glucose are represented in Supplementary Fig. S2. It is to be noted, in this connection, that our choice of 0.1 M glucose concentration used in photo-reforming experiments, came from preliminary measurements (Supplementary Fig. S3) that showed that further increase of glucose concentrations in the anolyte did not bring any significant advantage in terms of the achieved photocurrents.

Performed in parallel, measurements of incident photon-to-current conversion efficiency (IPCE) spectra of the WO_3_ photoelectrodes in the four supporting electrolytes revealed the spectra extending up to 480 nm with maxima close to 80% occurring in the 360–380 nm wavelength range (Fig. 2b). As already mentioned, because of the indirect optical transition in WO_3_^48^, the figures in the IPCE spectra are strongly dependent upon the SC film thickness, with the maximum that (vide infra*)* becomes shifted up to 400 nm for a ~3 μm thick WO_3_ film.

The extent of the photocurrent doubling resulting from the presence of glucose in the anolyte is directly illustrated by the IPCE plots (Fig. 2e) with the enhancement, with respect to the supporting electrolytes, close to factor 2. Like in the case of j-E plots (Fig. 2d), a larger increase of the IPCEs was observed in the solutions containing sulfates or chlorides, where peroxodisulfates (S_2_O_8_^2-^)^44,47^ or hypochlorous acid (HClO)^48^ may be formed, respectively.

An important insight into the nature of the products formed on a WO_3_ electrode during photo-oxidation of C1 organic molecules in a H_2_SO_4_ supporting electrolyte was provided^51^ by potentiodynamic on-line differential electrochemical mass spectrometry (DEMS) measurements employing continuous flow system. That study showed that although e.g., 0.01 M formic acid is photo-oxidized preferentially on the WO_3_ electrode this does not totally suppress the O_2_ formation that still accounts for about 10% of current efficiency.

**Fig. 3:**
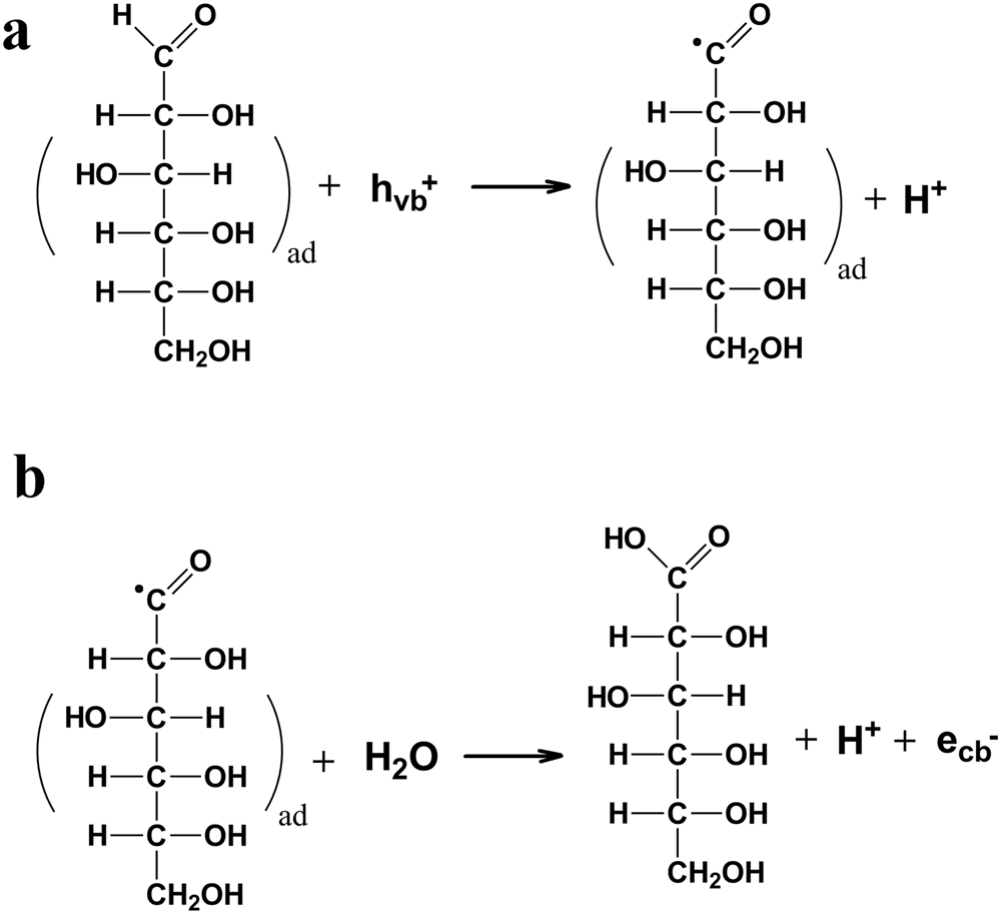
Suggested pathway for the photo-oxidation of glucose into gluconic acid. Sketch **a** represents a positive hole transfer to the adsorbed molecule of glucose followed by injection by the reactive intermediate of an electron into the CB of WO_3_, shown in sketch **b**, to form gluconic acid.

In comparison, larger (0.1 M) concentration of glucose employed in our experiments might suggest, at least at the initial stage of the photoreaction, less important contribution of radicals formed by oxidation of anions of the supporting electrolyte (such as HSO_4_^·^ in the case of 0.1 M NaHSO_4_) to the observed photocurrents. Consequently, the dominant pathway in the 2e^-^ oxidation of glucose to form gluconic acid might involve a direct transfer of a photogenerated positive hole to the adsorbed molecule of glucose (Fig. 3a) followed by injection by the reactive intermediate of an electron into the CB of WO_3_ to form gluconic acid (Fig. 3b).

However, assuming that further PEC reaction steps follow the desorption-re-adsorption mechanism^51^, the concentration of the intermediate (i.e., gluconic acid) leading to the formation of arabinose (corresponding to the transfer of 2e^-^) and of that of erythrose (transfer of additional 2e^-^) must be substantially lower. For this reason, it appears plausible that further stages of photoreaction (leading to arabinose and erythrose) follow an indirect pathway involving as intermediates the oxidation products of anions of the supporting electrolyte (cf. Fig. 2c).

### Identification of the glucose photo-oxidation products

**Fig. 4:**
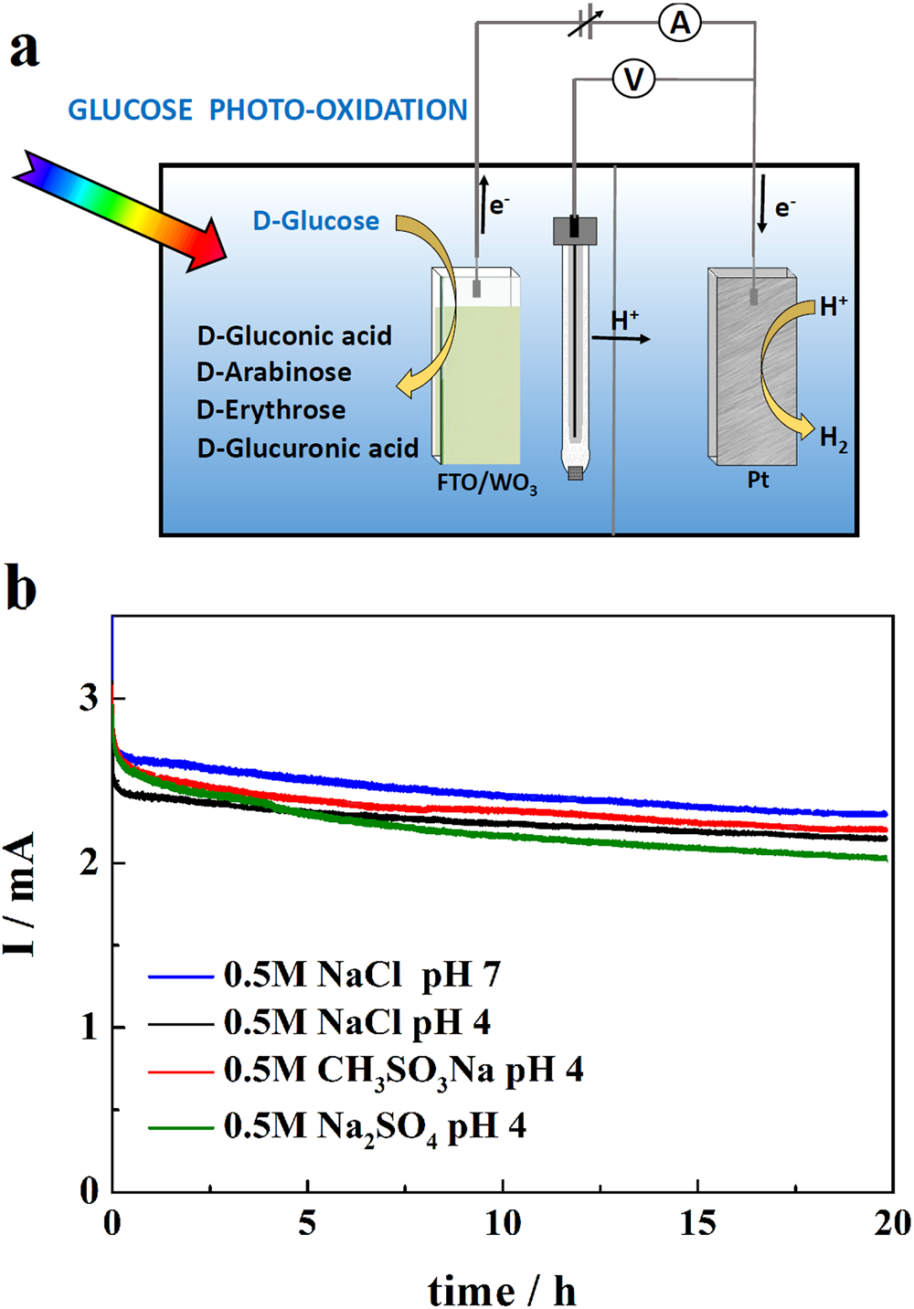
Photoelectrolysis of glucose. **a** Schematic of the photoelectrochemical cell used in glucose photo-reforming experiments. **b** The glucose photo-oxidation currents measured along 20 h electrolysis conducted at 1.23 V vs RHE in four different supporting electrolytes, using ~1.2 μm thick film WO_3_ electrodes irradiated with simulated AM 1.5 G sunlight. The Teflon cell used for those experiments contained 45 mL of solution in the anodic compartment and the illuminated area of the WO_3_ photoanodes was ~0.7 cm^2^.

To check the photostability of the WO_3_ photoanodes and, at the same time, probe glucose photo-oxidation products we conducted prolonged PEC experiments using a cell depicted in Fig. 4a. As shown in Fig. 4b, the photoanodes sustained a 20 h long photo-oxidation of 0.1 M solutions of glucose (present in the anolytes) in the four tested supporting electrolytes (i.e., chlorides, acidic sulfates, perchlorates and methanesulfonates) with a relatively small decline of the photocurrent that we assign to accumulation of the formed products within the pores inside the WO_3_ film. We also wish to notice, in this connection, that in contrast with the PEC behavior of the WO_3_ electrode in acidic sulfates electrolyte alone, characterized by a progressive photoanode deactivation associated with the formation of the surface peroxo species^44,47^, this did not occur when an equivalent amount of glucose was present in the anolyte. This observation might suggest that a part of the generated HSO_4_^·^ radical species, instead of forming peroxodisulfates, rapidly reacted with glucose.

**Fig. 5:**
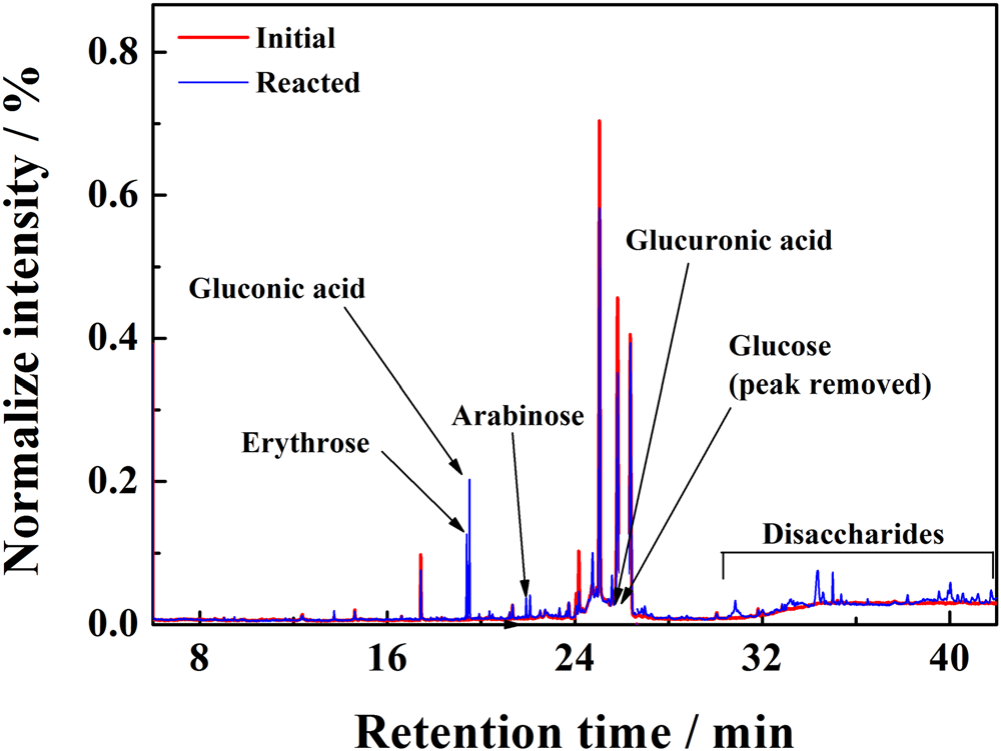
Chromatogram showing products formed over photoelectrolysis of 0.1 M glucose solution in 0.5 M NaCl electrolyte of pH 4. The red trace represents measurement taken before and the blue trace the one performed after 20 h long photoelectrolysis. For the electrolysis conditions see Fig. 4.

After completion of the 20 h long photoelectrolysis, products of glucose photoconversion present in the anolyte were detected and quantified by gas chromatography with mass spectrometry (GC-MS) analysis. The post photoelectrolysis compounds identified in the selected for analysis solutions of acidic sulfate and chloride electrolytes were: gluconic and glucuronic acids, arabinose and erythrose. Gluconic acid formed by 2e^-^ oxidation of glucose was by far the dominant product with the Faradaic yields (FY) of 16.5% and 14.6% achieved in acidic sulfate and chloride electrolytes, respectively (Supplementary Fig. S4). The FY of three other products remained in the range of 5% with the exception of erythrose produced with 7.7% FY in the sulfate electrolyte. A sample chromatogram in Fig. 5, shows that the photo-oxidized glucose solutions also contained disaccharides that could not be convincingly identified. For comparison we also included a chromatogram of a standard mixture of the principal products detected in the analytes (Supplementary, Fig. S5). Calibration curve was prepared by analyzing a series of standard samples containing compounds that are listed in Supplementary Table S1.

An interesting result was also provided by parallel total organic carbon (TOC) analyses (Supplementary, Fig. S6) that showed quite similar amounts of non-purgeable organic carbon (NPOC) species in fresh and in 20 h electrolyzed solutions. This suggests that, under conditions employed in our PEC experiments, the glucose was practically not converted into CO_2_ or into volatile organic compounds.

In the search for optimal photoelectrolysis conditions allowing to enhance the yield of glucose reforming products, we employed next less acidic supporting electrolytes (NaCl, Na_2_SO_4_ and CH_3_SO_3_Na) of pH 4. According to reports in the literature, the rate of glucose photo-reforming increases following increasing pH of the photocatalyst (TiO_2_) suspension^36^, and we hypothesized that a similar effect might be expected in the electrolytes employed for the PEC glucose oxidation. It is important to mention, in this context, that earlier investigations^52^ showed that glucose is converted into gluconate ions through hypochlorite oxidation in slightly alkaline solutions with highest yields obtained at pH 11.

**Fig. 6:**
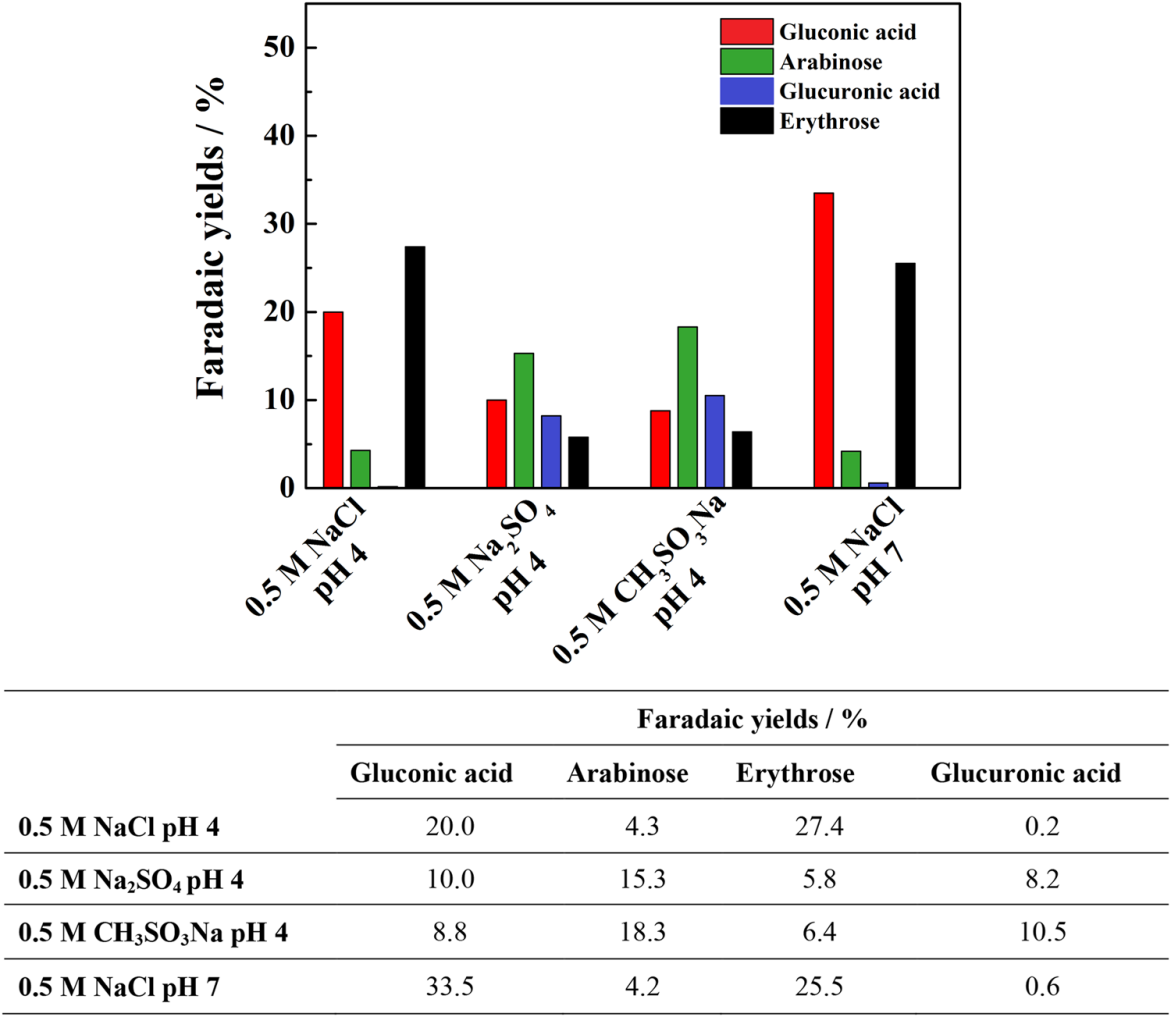
Amounts of photo-oxidation products formed over 20 h long photoelectrolysis of 0.1 M solutions of glucose performed in a series of supporting electrolytes. The electrolysis was conducted at 1.23 V vs RHE using WO_3_ and WO_3_/TiO_2_ photoanodes irradiated with simulated AM 1.5 G sunlight of 100 mW cm^−2^ intensity. The results are represented as Faradaic yields.

The PEC experiments using 0.5 M NaCl electrolyte of pH 4 with 0.1 mol L^−1^ of glucose (in the anolyte) allowed indeed the formation of substantially larger amounts of erythrose corresponding to FY of more than 27% and of gluconic acid with an about 20% FY. We recall, in this connection, that chlorine and hypochlorous acid are the dominant products generated on the WO_3_ photoanode during electrolysis of a 0.5 M NaCl solution^48^. As shown in Fig. 6, the distribution of the glucose photo-oxidation products determined after electrolysis performed in sulfate and methanesulfonate electrolytes of pH 4 was significantly different with, in particular, increased amounts of arabinose and of glucuronic acid, reflecting clearly the effect of anions of the supporting electrolytes. Comparison of the total Faradaic yields of the four analyzed products indicates the largest FY (of around 52%) determined in the 0.5 M NaCl (pH 4)/0.1 M glucose solution. Due to the small surface area of the WO_3_ photoanode (0.7 cm^2^) operating in relatively large volume (45 ml) of anolyte (cf. legend of Fig. 4) the extent of glucose conversion along 20 h long photoelectrolysis remained in the range of 15%.

**Fig. 7:**
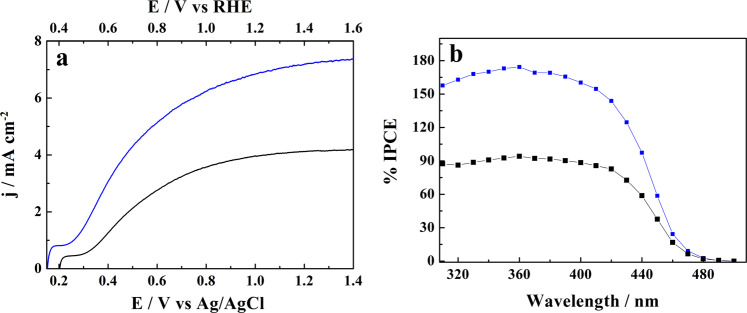
Effect of glucose addition upon photoelectrochemical characteristics of a ~ 3 μm thick film WO_3_ electrode. The measurements were performed in a 0.5 M NaCl of pH 4 solution alone and after addition of 0.1 mol L^−1^ of glucose. **a** Photocurrent densities vs imposed potential (j-E) plots recorded under simulated AM 1.5 G irradiation. **b** The corresponding IPCE spectra.

To assess the impact of the presence of glucose in the water splitting supporting electrolyte upon the efficiency of hydrogen generation in the PEC cell (see Fig. 4a), we used thicker, ~3 μm thick film photoanodes able to absorb more efficiently the blue portion of the solar spectrum close to the WO_3_ band edge. The j-E voltammograms recorded for the WO_3_ photoanode under simulated AM 1.5 G (of 100 mW cm^−2^ intensity) sunlight in the 0.5 M NaCl pH 4 electrolyte alone (black line) and in the solution containing 0.1 M glucose (blue line) (Fig. 7a) confirm the occurrence of a large extent of the photocurrent doubling resulting from the presence of glucose in the anolyte. At 1 V vs Ag/AgCl (~1.43 V vs RHE) high plateau photocurrents close to 6.5 mA cm^−2^ were attained. The corresponding IPCE plots (Fig. 7b) provide a clear explanation for the increased photocurrents displayed by the ~3 μm thick WO_3_ photoanode. Although the IPCEs measured at near UV wavelengths (up to 390 nm) are similar to those observed for a much thinner (1.2 μm thick) film photoanode (Fig. 2b), they are almost twice as large at a 440 nm visible wavelength, due to the substantially larger optical thickness of the WO_3_ film.

## Discussion

In this report, we demonstrated the ability of the semitransparent nanostructured WO_3_ photoanode to perform photo-reforming of glucose under simulated AM 1.5 G sunlight, with high photocurrent densities exceeding 6 mA cm^−2^ at 1.23 V vs RHE. Similar bias voltage may be provided by a number of single-junction PV cells enabling them to operate in a tandem device with the water splitting PEC cell. This opens the perspective to perform unassisted solar light-driven water splitting with high solar-to-hydrogen efficiency (STH) of more than 7%.

We note that the high achieved photocurrents are due in part to the preferential (in comparison with OER) photo-oxidation of glucose on WO_3_, accompanied, in addition, by the occurrence of the photocurrent doubling. In contrast with numerous other PEC and/or photocatalytic water splitting experiments in which biomass-derived compounds served only as sacrificial-kind agents to enhance hydrogen generation, our present work demonstrated photo-reforming glucose into value-added products. The obtained results also revealed the possibility of tuning the distribution of the glucose photo-oxidation products by the selection of anions of the supporting electrolyte and of its pH. In fact, a comparison of the Faradaic yields corresponding to photo-oxidation of glucose in NaCl electrolytes of pH 2 and pH 4 (cf. Supplementary Fig. S4 and Fig. 6 in the full text) shows, in the second case, a substantial increase of FY of both gluconic acid (~20%) and of erythrose (~27%). Considering the FY of the analyzed glucose photo-reforming products (gluconic and glucuronic acids, arabinose and erythrose), the NaCl electrolyte of pH 4 appears as the most effective with the total FY of around 52%. It is important to recall, in this connection, that chlorine and hypochlorous acid are the principal photo-oxidation products at the WO_3_ photoanode in the 0.5 M NaCl electrolyte^48^. We also note the close similarity between the distribution of the glucose photo-reforming products identified in CH_3_SO_3_Na and Na_2_SO_4_ electrolytes of pH 4. Since in the former case, it is the highly reactive CH_3_SO_3_^·^ radical (unable to form a dimer)^50^ that, apparently, mediates photo-oxidation of glucose we hypothesize that in the sulfate supporting electrolyte a similar role is played by the SO_4_^**-**^^·^ radical anion.

Following earlier reports in the literature^36,52^ indicating that the rate of glucose oxidation increases with moving the solution pH in alkaline direction, we attempted to perform glucose photo-reforming using less acidic electrolytes. Since the range of thermodynamic stability of crystalline monoclinic WO_3_ in aqueous solutions extends from strong acids to pH 4.6^53^, in order to minimize the possibility of photo-corrosion we decided to cover the outer surface of the WO_3_ film with a thin ~0.4 μm thick deposited layer of TiO_2_ nanoparticles (P25 from Ovonics). Unlike WO_3_, TiO_2_ is stable both in acidic as well as in alkaline solutions. Thus formed hybrid WO_3_/TiO_2_ electrode was tested in a 0.5 M NaCl (pH 7)/0.1 M glucose solution, irradiated from the rear side (i.e., through the FTO substrate) with simulated 1 sun AM 1.5 G light. Despite slightly diminished light transmission by FTO, the IPCEs attain a maximum of ~160% at 360 nm (Supplementary Fig. S7a) with still observable photoresponse extending up to 480 nm. Interestingly, comparison with the IPCEs recorded in the same configuration for an identical WO_3_ film electrode without a TiO_2_ overlayer, shows at 410–460 nm wavelengths a clearly increased values for the hybrid WO_3_/TiO_2_ electrode. Since anatase TiO_2_ (the dominant form present in P25) does not absorb (and largely transmits) wavelengths above 400 nm, we hypothesize that the increased IPCEs may be due to the dispersion/reflection at the WO_3_ – TiO_2_ interface of a certain fraction of visible light unabsorbed by WO_3_. Consequently, the j-E plots measured for the WO_3_/TiO_2_ electrode under the rear-side AM 1.5 G (of 100 mW cm^−2^ intensity) irradiation (Supplementary Fig. S7b) attain high saturation photocurrents of ~ 3.8 mA cm^−2^ comparable with those observed in the solutions of lower pH under the solution-side illumination (cf. Fig. 2a). The aforementioned conditions were subsequently employed for prolonged 20 h long photoelectrolysis of a 0.5 M NaCl (pH 7)/0.1 M glucose solution, that revealed a large increase of the amount of formed gluconic acid to reach ~34% FY, about 65% larger than the FY determined after experiment conducted in the analogous solution of pH 4 (Fig. 6).

An additional photoelectrolysis employing a 0.5 M NaCl/0.1 M glucose anolyte of pH 7, that involved product analysis also after 5 h and 10 h along the 20 h long PEC experiment, showed regular increase of the amounts of formed products (gluconic acid, glucuronic acid, arabinose and erythrose) over 5 h, 10 h up to 20 h periods (Supplementary Fig. S8).

In summary, we have shown that a PEC device consisting of a nanostructured WO_3_ photoanode and of a metallic cathode operates in a sustainable way reforming glucose into valuable products and producing hydrogen in a separate cell compartment. By using a WO_3_ photoanode having larger optical thickness to match the penetration depth of blue light photons, we demonstrated under AM 1.5 G sunlight at 1.23 V_RHE_ photocurrents exceeding 6 mA cm^−2^. The latter value translates into expected solar-to-hydrogen efficiency above 7% to be attained in the tandem cell. For comparison, we collected together (Supplementary Table S2) the PEC performances of different WO_3_ photoanodes reported in the papers cited in the present article.

The prolonged photoelectrolysis runs confirmed the absence of any deactivation of the WO_3_ electrode by the formed products. Our investigation of the photo-reforming process demonstrated the versatility of the WO_3_ photoanode able to direct the distribution of the glucose oxidation products through the choice of appropriate electrolyte. Further improvements in the selectivity of the photo-reforming process may involve the use of mixtures of supporting electrolytes, i.e., combinations of different anions acting as intermediates in the photo-oxidation reactions on the WO_3_ electrode.

## Methods

### Reagents

Sodium tungstate dihydrate, poly(ethylene)glycol (PEG) 300 (used to prepare WO_3_ films), TiO_2_ P25 (containing ca. 75% of anatase and 25% of rutile (Ovonics), poly(vinylidene-fluoride), dimethylformamide, methanesulfonic acid (99.5%), perchloric and sulfuric acids, sodium chloride and glucose were purchased from Sigma-Aldrich; hydrochloric acid was purchased from Chempur. The solutions used in electrochemical measurements were prepared with Mili-Q water. The reagents employed to prepare samples for GC-MS and TOC analyses are listed in the Supplementary Methods.

### Preparation and structural characterization of the WO_3_ photoelectrodes

The mesoporous WO_3_ films used in this work were formed on conductive glass substrates through doctor blade layer-by-layer deposition of a colloidal solution of tungstic acid, containing an organic structure-directing agent – low molecular weight poly(ethylene glycol) PEG 300. The formation of a complex between tungstic acid and the hydrophilic PEG delays the formation of fully crystallized monoclinic tungsten trioxide until heating to ~ 500 °C. Preparation of the precursor solution followed the earlier described original procedure^43^. Conductive glass plates (Sigma-Aldrich, resistance 7 Ω/square) bearing a 0.9 μm thick overlayer of F-doped SnO_2_ (FTO) were used as substrates. The WO_3_ films were formed by three up to eight consecutive applications of the precursor solution each followed by annealing in flowing oxygen at 550 °C for 30 min. The final thickness of such films (determined from the cross-sectional scanning electron microscopic, SEM, images) varied between ~1.2 to about 3 μm.

To obtain hybrid WO_3_/TiO_2_ electrodes, we deposited on ~1.2 μm thick WO_3_@FTO films ca 0.4 μm thick TiO_2_ overlayers^25^. The TiO_2_ film was formed by one application from the prepared suspension (1 g TiO_2_ powder in 20 ml DMF with 0.4 g poly(vinylidenefluoride)) onto WO_3_ layer. Then it was dried in air for 30 min., later for solvent evaporation at 100 °C for 40 min and followed by annealing at 500 °C for 1 h in oxygen flow.

SEM imaging was performed using a Carl Zeiss Auriga Cross-Beam workstation. The microscope was equipped with a Gemini electron column with Energy Selective Backscattered (ESB) detector.

X-ray powder diffraction (XRD) measurements were performed on the as-grown samples using a Siemens D500 diffractometer equipped with a high-resolution semiconductor Si:Li detector using Cu Kα radiation (U = 40 kV, I = 30 mA). The powder diffraction patterns were measured in a θ/2θ scanning mode with a step of 0.02° and counting time of 10 s by step.

Raman spectra were acquired with the Alpha 300 M + confocal microscope (Witec) equipped with a motorized stage. For analyses we used a 532 nm laser line delivered to the microscope through a single-mode optical fiber. The laser power focused at the sample did not exceed 7 mW. The backscattered Raman signal was collected through a 50× long working distance objective (NA = 0.42), and passed through a photonic optical beam to a lens-based spectrometer (Witec UHTS 300, f/4 aperture, focal length 300 mm) coupled with a back-illuminated Andor iDUS 401 detector thermoelectrically cooled to −60 °C. The spectra were collected in the range of Raman shifts from 100 to 1000 cm^−1^ with the use of an 600 mm grating. For each sample Raman mapping was conducted on a 50 × 50 μm^2^ area with a 1 μm step (totaling to 2500 spectra) and a 0.3 s acquisition time. The spectra were postprocessed (background subtraction and cosmic-ray removal) with the Project FIVE software (Witec). For all samples, the spectra in the mapped area were homogeneous. Consequently, an average over 2500 spectra was made for each sample.

### Photoelectrochemical measurements

The photocurrent-potential (j-E) plots and incident photon-to-current conversion efficiencies (IPCEs) were measured in a Teflon cell equipped with a quartz window, by illuminating the WO_3_ electrode either from the side of the solution/film interface or, in some cases, through the FTO substrate. The simulated AM 1.5 G (of 100 mW cm^−2^ intensity) illumination was obtained from an Oriel 150 W solar simulator. In those measurements, the exposed WO_3_ electrode surface area was 0.28 cm^2^. The measurements were performed in a three-electrode configuration, with a WO_3_ photoanode a platinum grid counter electrode and a silver/silver chloride (Ag/AgCl) in sat. KCl (E = 0.197 vs SHE) as a reference electrode. The potentials are also quoted versus reversible hydrogen electrode (RHE) in the same solution. To obtain j-E plots, the potential of the WO_3_ electrode was swept from the open circuit potential of ~0.40 V_RHE_ (in the solutions containing glucose) to 1.6 V_RHE_ at a rate of 10 mV sec^−1^ using a CH Instrument - CHI 660E electrochemical work-station. The IPCE spectra of the WO_3_ photoanodes were determined using light from a 150 W xenon lamp passing through a photoelectric spectrometer (Instytut Fotonowy) featuring monochromator with a bandwidth of 10 nm. The absolute light intensity was measured with a model OL 730-5 C UV-enhanced silicon detector Gooch&Housego.

Preliminary long-term photoelectrolyses of the anolyte solutions containing glucose, intended to check the photostability of the photoanode, were carried out in a large volume quartz cell containing 400 mL of solution with anodic and cathodic compartments separated by a Nafion membrane. In those tests, a total surface area of the WO_3_ films deposited on FTO (~1.6 cm^2^) was exposed to the electrolytes. Further 20 h-long experiments intended for identifying glucose photo-reforming products were performed in a smaller Teflon cell with ca 45 mL of anolyte and ~0.7 cm^2^ illuminated WO_3_ surface area.

### Identification of the glucose oxidation products

To identify the products of glucose oxidation GC-MS-QP2010 Ultra gas chromatograph (Shimadzu) interfaced with a single quadrupole mass spectrometer was used. The instrument was equipped with AOC-5000 autosampler (Shimadzu) in the liquid-injection mode featuring a 10 µl syringe. The syringe was washed three times before and after each injection with ethyl acetate and then with hexane. The analytes were separated using a capillary column ZB-5MSPlus (Phenomenex): 30 m x 0.25 mm, 0.25 µm stationary phase film. The column head pressure was 26.7 kPa, the total flow of the carried gas was 9.5 ml/min, column flow 0.68 ml/min (30 cm/sec) and the purge flow was 2 ml/min. The linear velocity flow control mode was used and the split ratio was 10. The temperatures of injector, ion source and the mass spectrometer transfer line were 300 °C. The following temperature program was used: initially 50 °C held for 2 min, then linear increase at the rate of 10 °C/min to 225 °C kept for 10 min, then linear increase at the rate of 15 °C/min to 280 °C, held for 10 min.; the total analysis time was 43 min. Mass spectrometer was equipped with the electron impact (EI) ion source and operated in the scan mode in the mass range 45-500 m/z (scan time 150 ms), the solvent cut was 6 min. For the quantitative analysis of the formed products (more concentrated samples), the glucose peak was removed from the chromatogram by turning off the mass spectrometer between 25.9 and 26.3 min to avoid oversaturating the detector and damaging the ion source. GCMSsolution 2.53 (Shimadzu) program was used for data acquisition and processing.

Total organic carbon (TOC) analysis was quantified with TOC-5050A analyzer (Shimadzu) equipped with the ASI-5000A autosampler (Shimadzu) in order to check the possible formation of volatile glucose oxidation products.

### Sample preparation

a) for GC/MS analyses

Aliquots of the reaction solution were evaporated to dryness at 60 °C under a gentle stream of N_2_ and derivatized prior to the GC/MS analysis.

When the reaction solution contained concentrated mineral acids (H_2_SO_4_ or HCl), the samples were neutralized before evaporation to avoid the mineralization of glucose and other saccharides that was observed during preliminary experiments. For the quantitative analysis of glucose, the reaction solution was diluted twenty times with DI water to the total volume of 1 ml; 5 µl of this solution was evaporated to dryness at 60 °C under a gentle stream of nitrogen. For the quantification of the products formed, 2 µl of the reaction solution were dried without any prior dilution.

The dried samples were derivatized according to the described procedure that was optimized for saccharides and uronic acids^54^.

Briefly, this tree-step derivatization protocol involved mercaptalation with ethanethiol and two silylation steps with BSTFA/TMCS (99:1, v/v).

GC measurement uncertainties for products concentrations were calculated from several injections and are presented in Supplementary Table S3.

b) for Total organic carbon

Firstly, the instrument was calibrated with the standard solutions of glucose in DI water with concentrations between 4 and 36 mg_TOC_ × L^−1^; the squared linear coefficient of determination for the glucose calibration curve (R^2^) = 0.9994 was obtained.

The aliquots of the reaction solution were diluted approx. five hundred times with DI water before the analysis to adjust the total organic carbon (TOC) concentration between 20 and 35 (mg × L^−1^). Afterward, each sample was filtered with a single-use PTFE syringe filter (pore size 0.22 µm) and 4 ml of the filtered solution was placed in the instrument autosampler. Subsequently, 50 µl of 2 M HCl was added via the autosampler and each sample was sparged with oxygen for 2 min before injection. The injection volume was 21 µl and each sample was injected into the instrument three times.

## Supplementary information

Supplementary Information

## Data Availability

The authors declare that all data supporting the findings of this study are available within the article and its supplementary information files, and from the corresponding author on reasonable request.
