# Supplementary material for: Photoelectrocatalytic hydrogen generation coupled with reforming of glucose into valuable chemicals using a nanostructured WO3 photoanode
Source: Commun Chem. 2022 Oct 13;5:125. doi: 10.1038/s42004-022-00745-w (PMC9814346; doi:10.1038/s42004-022-00745-w)
Supplement: Supplementary file 1 — Supplementary Information [file 42004_2022_745_MOESM1_ESM.pdf]

Supplementary Information

Photoelectrocatalytic hydrogen generation coupled with reforming of glucose into valuable chemicals using a nanostructured WO_3 photoanode

Katarzyna Jakubow-Piotrowska[1]*, Bartłomiej Witkowski[2], Jan Augustynski[1]*

[1] Centre of New Technologies, University of Warsaw, S. Banacha 2c, 02-097, Warsaw, Poland.

[2] Faculty of Chemistry, University of Warsaw, Pasteura 1, 02-093 Warsaw, Poland.

* J. Augustynski

E-mail: j.augustynski@cent.uw.edu.pl

* K. Jakubow-Piotrowska

E-mail: k.jakubow@cent.uw.edu.pl

Centre of New Technologies, University of Warsaw, S. Banacha 2c, 02-097, Warsaw, Poland

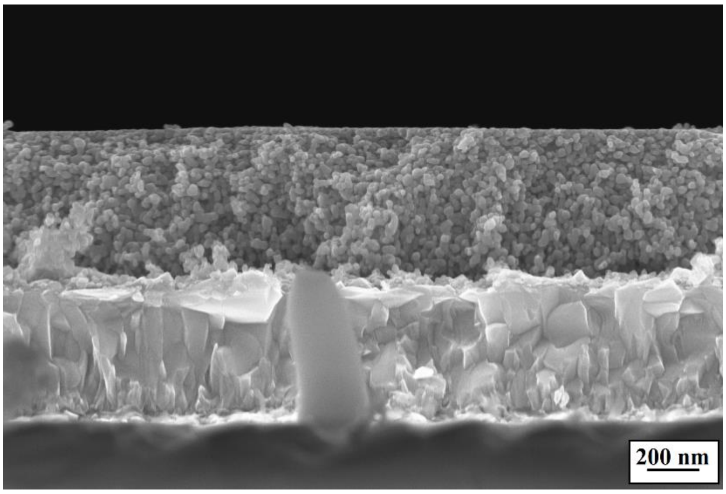

Fig. S1 Cross-sectional SEM image of a ~ 0.4 μm thick WO_3 film deposited on FTO using the sol-gel method and annealed at 550°C.

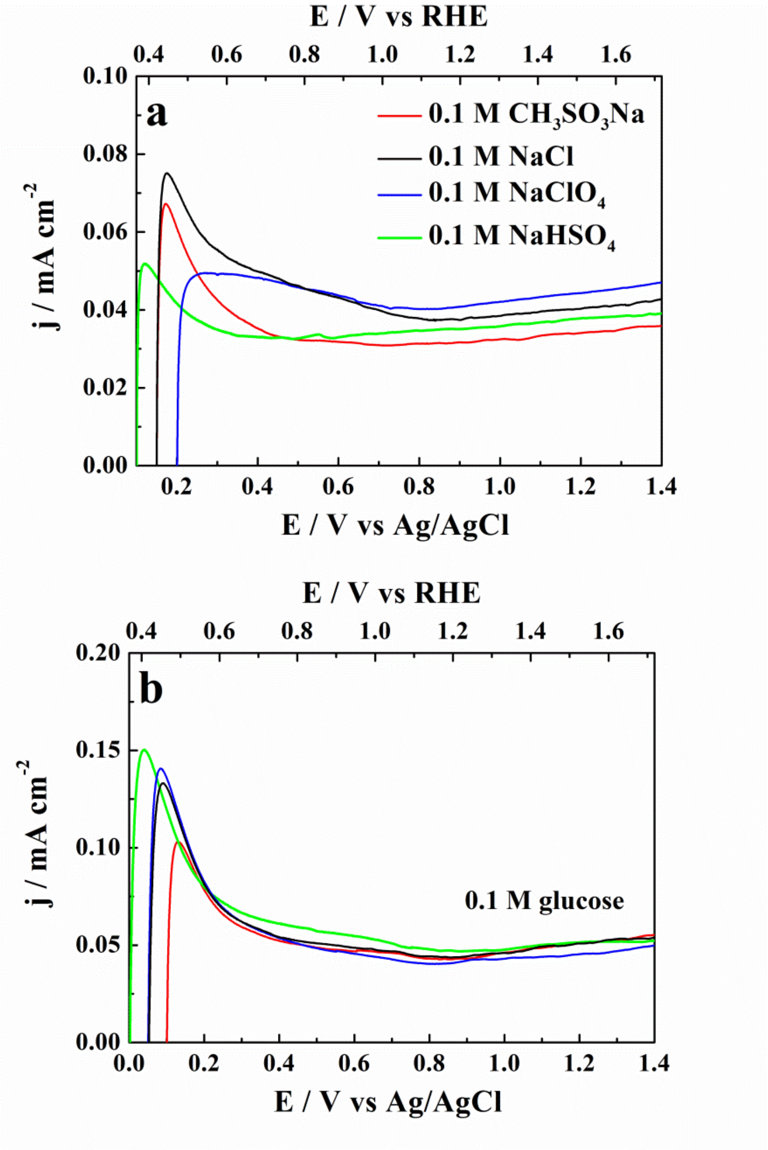

Fig. S2 a, Photocurrent densities vs imposed potential (j-E) plots for a ~ 1.2 µm thick film WO_3 electrode in a series of 0.1 M supporting electrolytes acidified to pH 2 and **b**, also with added 0.1 mol/L of glucose in the absence of illumination.

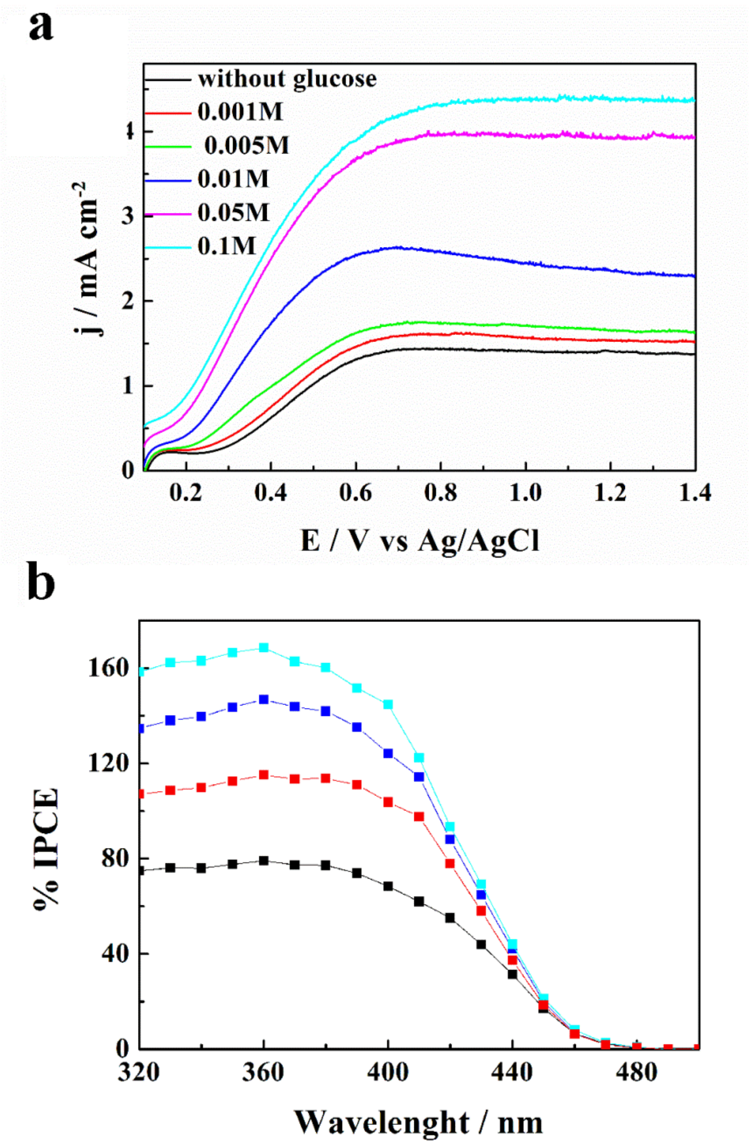

Fig. S3 a, Photocurrent – potential (j-E) curves determined for a ~ 1.2 μm thick WO_3 electrode in a series of 0.1M $NaHSO_4$ solutions of pH 2 containing increasing concentrations of glucose. Measurements were performed under simulated AM 1.5G (of 100 mW cm^{-2} intensity) solar illumination. **b,** The corresponding IPCE spectra.

Supplementary Methods

Reagents

D-(-)-arabinose (≥98.0%), D-glucuronic acid (≥97.0%), D-gluconic acid (≥97.0%),D-(+)-galacturonic acid (≥97.0%), D-ribonic acid (≥95.0%), D-(+)-maltose monohydrate (≥99.0%), D-(-)-ribose (≥99.0%), D-(-)-erythrose (≥99.0%), D-xylonic acid (≥95.0%), anhydrous lactose (Pharmaceutical Secondary Standard), trifluoroacetic acid (≥99.0%), ethanetiol (≥97%) were obtained from Merck (Schnelldorf, Germany). Anhydrous pyridine (reagent grade), N,O-Bis(trimethylsilyl)trifluoroacetamide containing 1% of trimethylsilyl chloride (BSTFA+1%TMCS, GC-derivatization grade) were obtained from Chemat Adam Taszner (Grańsk, Poland). Hexane (≥99.0% Ultra-grade for organic residue analysis) was obtained from Avantor Performance Materials (Gliwice, Poland). Deionized (DI) water (18 MΩ × cm^{-1}) was prepared using Direct - Q3 Ultrapure Water System (Millipore). Ultra-high purity gasses: oxygen (used in the total organic carbon analyzer), helium (GC/MS carried gas) and nitrogen (for sample evaporation) were supplier by Multax (Stare Babice, Poland).

Analysis of standards and calibration curve (GC/MS)

Standards were prepared by evaporating different volumes of the standard solutions in DI water that contained a mixture of the five analytes. Each sample was derivatized as described in Methods.

Name	Retention time (min)	m/z or scan	Mass range (µg)	Obtained linear coefficient of determination (R^2)
Compounds identified in the reacted samples and quantified				
Erythrose	19.2	135	0.5 - 9	0.9938
Gluconic acid	19.3	319		0.9930
Arabinose	21.7	scan		0.9945
Glucuronic acid	25.8			0.9996
Glucose *(substrate)*	26.0			0.9943
Additional standards, not detected in the reacted samples				
Xylose	21.6	-		
Ribose	20.6			
Ribonic acid	17.9			
Galacturonic acid	24.8			
Xylonic acid	18.1			
Maltose	39.7, 40.75, 41.55			
Lactose	42.2			

Table S1 List of analyte and results of the instrument calibration.

As listed in Table S1, additional commercially available standards were analyzed but because they were not detected in the reacted samples the calibration was carried out only for glucose (precursor), erythrose, arabinose, gluconic acid and glucuronic acid.

Sample chromatogram of the standard mixture of the analytes quantified (concentration ca. 5 µg of each) is presented in Fig. S5.

As presented in Fig. S4, under the experimental conditions, glucuronic acid and glucose were sufficiently separated but it was impossible to obtain a baseline separation for these two compounds under the GC/MS analysis. Overlapping peaks for gluconic acid and erythrose were observed due to co-elution. For this reason, quantification of these two analytes was carried out using characteristic fragmentation ions (Table S1).

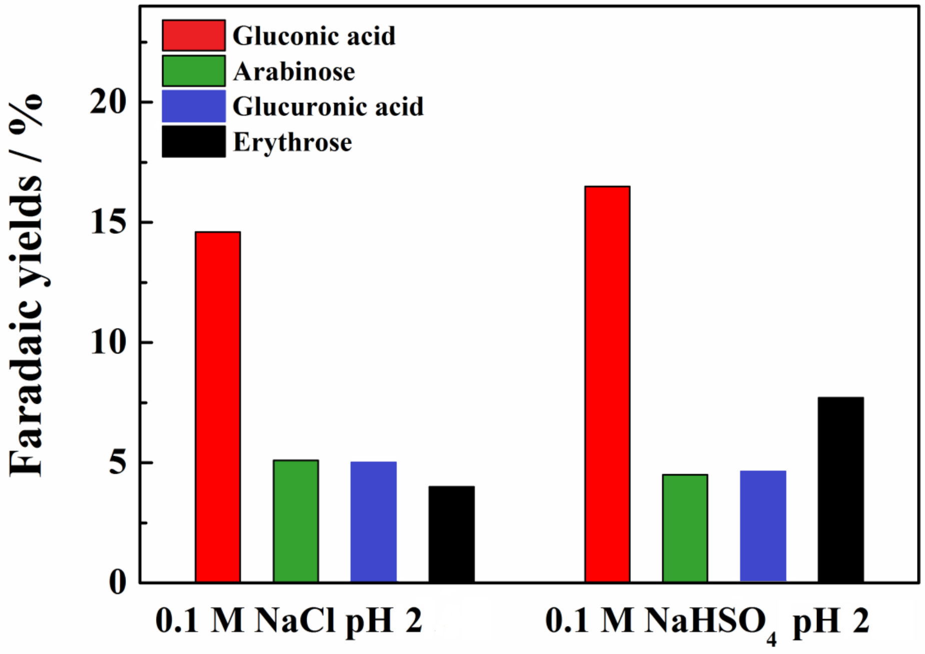

	Faradaic yields / %			
	**Gluconic acid**	**Arabinose**	**Erythrose**	**Glucuronic acid**
**0.1 M NaCl pH 2**	14.6	5.1	4.0	5.0
**0.1 M $NaHSO_4$ pH 2**	16.5	4.5	7.7	4.6

Fig. S4 Faradaic yields of glucose photo-oxidation products determined after 20 h long photoelectrolysis of 0.1 M solutions of glucose in 0.1 M NaCl and NaHSO4 electrolytes acidified to pH 2. The WO3 electrodes were polarized at 1.23 V vs RHE and irradiated with simulated AM 1.5G sunlight of 100 mW cm^{-2} intensity.

Material	Electrolyte	Photocurrent	Potential	Light source	Ref.
WO_3	0.33M H_2SO_4	~ 0.9 mA/cm^2 for 3µm thick electrode	at 1.2 V vs SCE	AM 1.5G intensity (100 mW cm^{-2})	35
WO_3	0.1M KH_2PO_4 buffer pH 7	~ 0.5 mA/cm^2	-	AM 1.5G, (100 mW cm^{-2})	45
WO_3	a) 1M HCl<br>b) 1M H_2SO_4<br>c) 1M $HClO_4$	a) ~ 1.5 mA/cm^2<br>b) ~ 1.25 mA/cm^2<br>c) ~ 1.0 mA/cm^2	at 1.2 V vs NHE	simulated AM 1.5G solar illumination	46
WO_3	a) 0.1M CH_3COOH pH 3<br>b) 0.1M CH_3COONa pH 5<br>c) 0.1M NaCl pH 1,3,5	a) ~ 0.6 mA/cm^2<br>b) ~ 1.2 mA/cm^2<br>c) ~ 1.0-1.2 mA/cm^2	at 0.9 V vs Ag/AgCl	AM 1.5G intensity (100 mW cm^{-2})	47
WO_3	a) 0.5M H_2SO_4<br>b)0.5M H_2SO_4 with 0.01 M CH_3OH	a) ~ 0.8 mA/cm^2<br>b) ~ 1.8 mA/cm^2	at 1.2 V vs RHE	UV light illumination	51
WO_3	a) 0.5M NaCl pH 4<br>b) 0.5M NaCl pH 4 with 0.1 M glucose<br>c) 0.5M NaCl pH 4<br>d) 0.5M NaCl pH 4 with 0.1 M glucose	a) ~ 2 mA/cm^2 for 1.2µm thick electrode<br>b) ~ 3.5 mA/cm^2<br>c) ~ 3.5 mA/cm^2 for 3 µm thick electrode<br>d) ~ 6.5 mA/cm^2	at 1.0 V vs Ag/AgCl	AM 1.5G intensity (100 mW cm^{-2})	this work

Table S2 PEC performances of WO_3 photoanodes used in this work compared with those reported by other authors cited in the present article.

	RSD / %			
	**Gluconic acid**	**Arabinose**	**Erythrose**	**Glucuronic acid**
**0.1 M NaCl pH 2**	4.0	3.0	8.0	5.0
**0.1 M $NaHSO_4$ pH 2**	7.0	5.0	6.0	5.0
**0.5 M NaCl pH 4**	1.1	1.0	0.5	1.4
**0.5 M Na_2SO_4 pH 4**	2.3	0.6	1.8	3.7
**0.5 M CH_3SO_3Na pH 4**	3.8	3.3	3.1	2.5
**0.5 M NaCl pH 7**	3.0	3.8	2.7	2.7

Table S3 Uncertainties related to GC determined concentrations of glucose photo-reforming products listed in Figures 6 and S4. Presented as Relative Standard Deviations, the RSD values were calculated from several analyte injections.

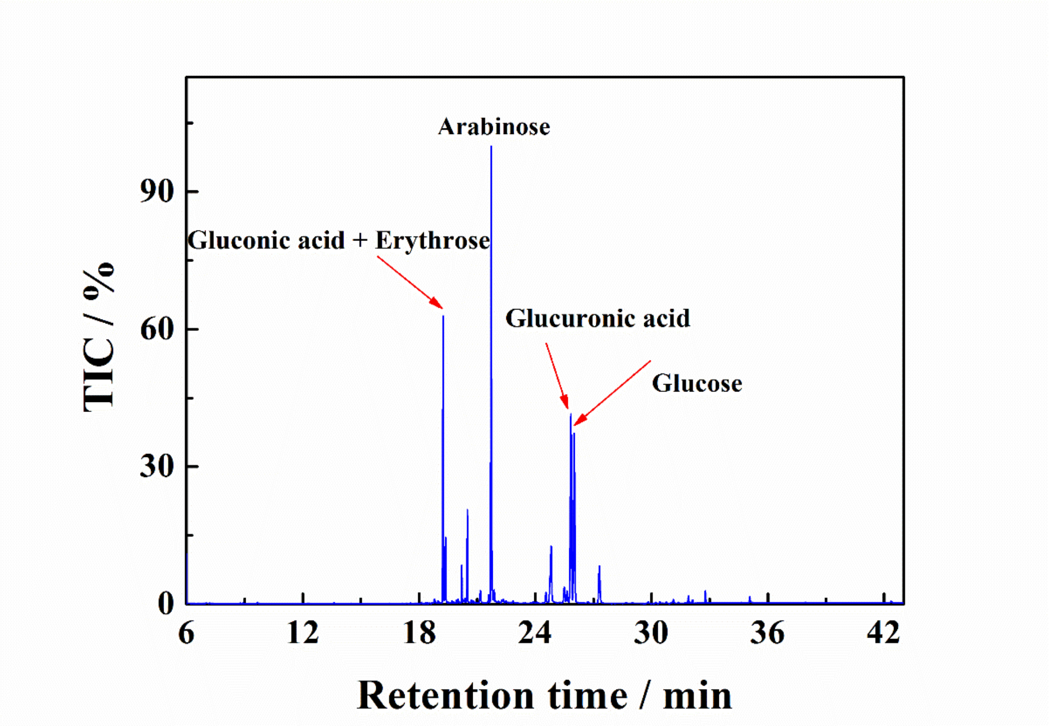

Fig. S5 Representative chromatogram of the standard mixture of principal organic compounds (approx. 5 µg of each) involved in the photo-reforming of glucose.

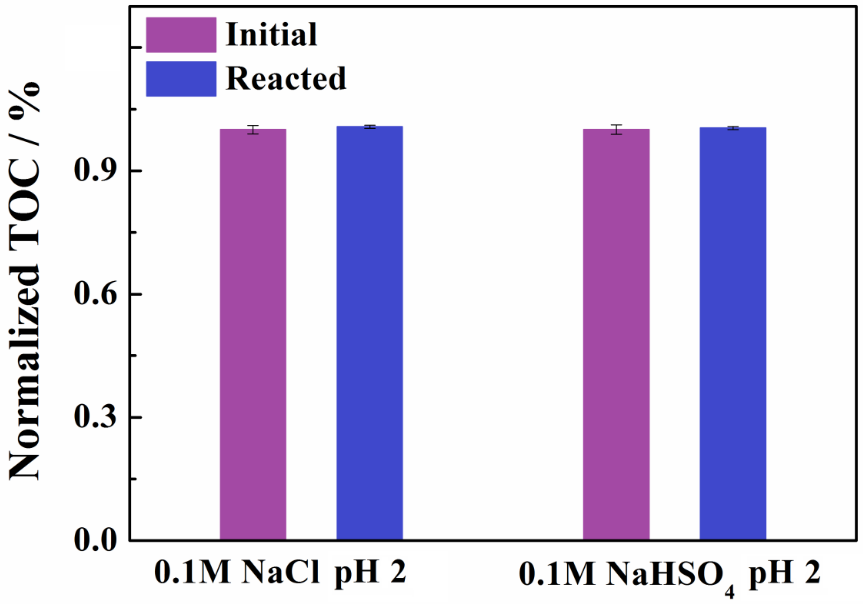

Fig. S6 Relative amounts of non-purgeable organic carbon (NPOC) measured for fresh and reacted samples. The glucose photoelectrolysis conditions were those specified in legend of **Fig. S4**. Uncertainty bars represent 2σ values from triplicate injection into the instrument

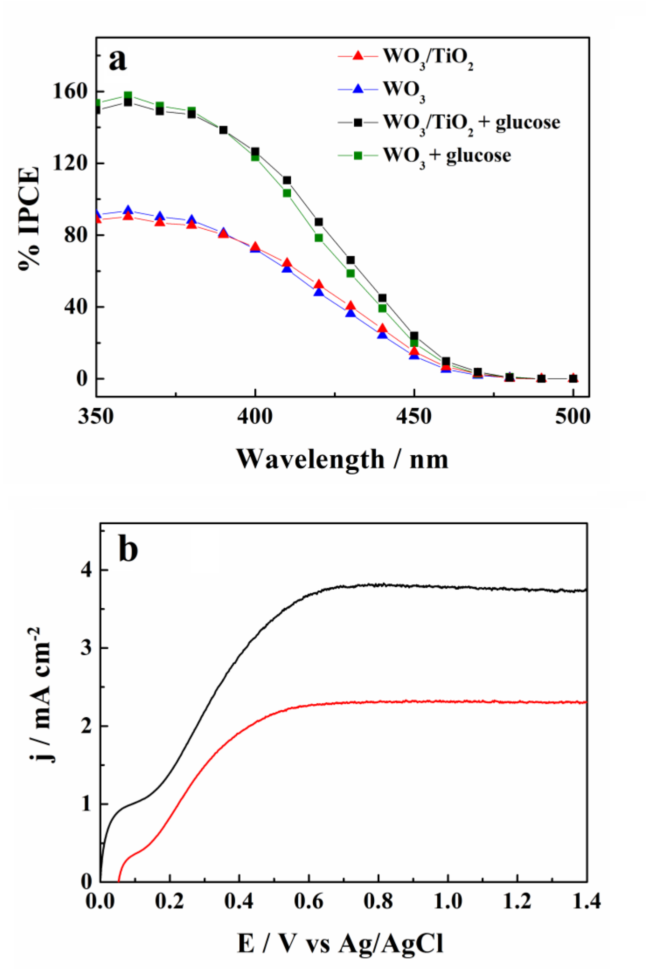

Fig. S7 Photoelectrochemical performance of a WO_3/TiO_2 electrode compared with that of a WO_3 electrode alone. Both electrodes consisted of ~ 1.2 μm thick WO_3 films with (for the first case) an ~ 0.4 μm thick TiO_2 overlayer and were illuminated from the rear side, i.e., through the FTO substrate. The measurements were conducted in a 0.5 M NaCl supporting electrolyte of pH 7 containing 0.1 mol L^{-1} of glucose. **a,** The incident photon-to-current conversion efficiency (IPCE) spectra measured at 1.23 V vs RHE. **b,** Photocurrent densities vs imposed potential (j-E) plots recorded under simulated AM 1.5G irradiation.

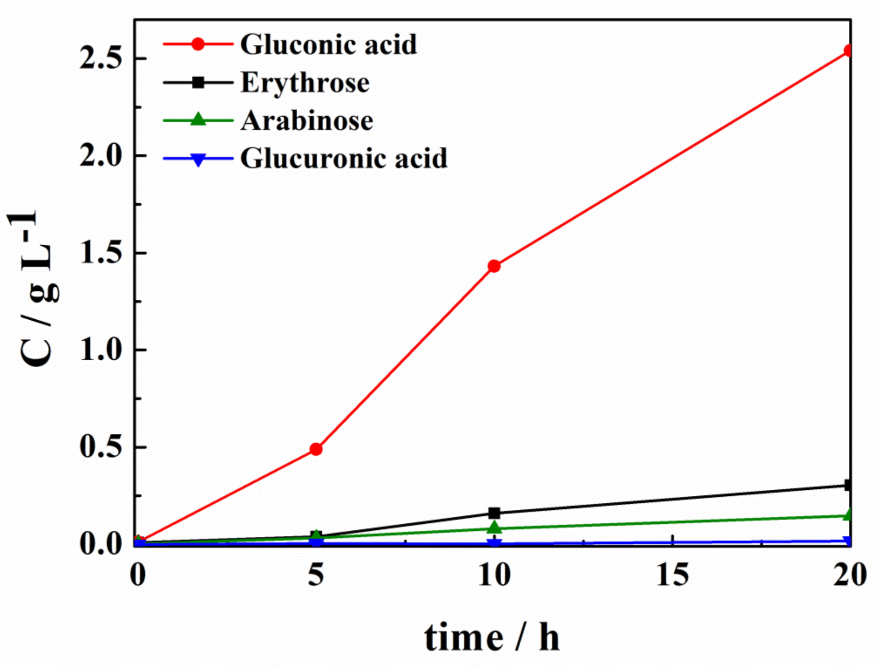

Fig. S8 Product analysis after 5, 10 and 20 hours long PEC experiment that employed a 0.5 M NaCl/0.1 M glucose solution of pH 7.
